# Is Humane Slaughter Possible?

**DOI:** 10.3390/ani10050799

**Published:** 2020-05-05

**Authors:** Heather Browning, Walter Veit

**Affiliations:** 1 School of Philosophy, Australian National University, Canberra, ACT 0200, Australia; 2 School of History and Philosophy of Science, University of Sydney, Camperdown, NSW 2006, Australia; wrwveit@gmail.com

**Keywords:** slaughter, humane, welfare, harm, shape of a life

## Abstract

**Simple Summary:**

When looking at the welfare of farmed animals, it is important to also consider the conditions at the end of their lives. How animals are transported and slaughtered can have a large impact on their lifetime welfare. Though most work focusses on reducing the pain and suffering experienced during slaughter, we argue that to be humane, slaughter must not create any kind of harm to the animal. As death itself is harmful to welfare—due to depriving the animal of future positive experiences—slaughter can never be truly humane. Furthermore, the order in which an animal experiences positive and negative events has an impact on welfare, and since slaughter places suffering at the end of life, it is even more harmful. Although these considerations mean that no slaughter can ever be completely humane, it is still important to continue research to improve practices so that as long as it continues, harms to welfare are minimised as much as possible.

**Abstract:**

One of the biggest ethical issues in animal agriculture is that of the welfare of animals at the end of their lives, during the process of slaughter. Much work in animal welfare science is focussed on finding humane ways to transport and slaughter animals, to minimise the harm done during this process. In this paper, we take a philosophical look at what it means to perform slaughter humanely, beyond simply reducing pain and suffering during the slaughter process. In particular, we will examine the issue of the harms of deprivation inflicted in ending life prematurely, as well as shape of life concerns and the ethical implications of inflicting these harms at the end of life, without the potential for future offsetting through positive experiences. We will argue that though these considerations may mean that no slaughter is in a deep sense truly ‘humane’, this should not undermine the importance of further research and development to ensure that while the practice continues, animal welfare harms are minimised as far as possible.

## 1. Introduction

One of the biggest ethical issues in animal farming, husbandry, and agriculture more generally, is the welfare of animals during the process of slaughter. Over 65 billion land animals across the globe are killed for food each year [1] a number that is only rising. Although most work to date in animal welfare science has concentrated on improving the living conditions of animals, there is an increasing focus on finding humane ways to transport and slaughter animals, in order to minimise the harm done during this process. An example of a significant change of this type has been the introduction of the use of stunning methods prior to the actual act of killing, reducing the pain and suffering caused in these final moments, and thus constituting a vast improvement in welfare [2]. The introduction of numerical scoring systems for animal welfare in abattoirs, primarily based on assessing the effectiveness of stunning, is another example of a large-scale change leading to welfare improvement [3]. In this paper, we go beyond a call for simply reducing pain and suffering during the slaughter process and take a more philosophical perspective on what it means to perform slaughter humanely.

We need to be clear here on what we mean by ‘humane’. In general, it is taken to mean something like ‘minimising the harm inflicted’ (see e.g., [2]) or ‘avoiding unnecessary suffering’ [4]. The RSPCA (Royal Society for the Prevention of Cruelty to Animals) describes humane slaughter as an animal being “either killed instantly or rendered insensible until death ensues, without pain, suffering or distress” [5], while the American Veterinary Medical Association Guidelines for humane slaughter describe it as “minimizing (and, where possible, eliminating) anxiety, pain, and distress associated with terminating the lives [of animals]” ([6], p.5). Here, we take a slightly stronger position and argue that for a practice to be truly humane, it must not cause any (or minimal) harm to welfare, which includes harms of deprivation. The importance of this focus on overall harm to welfare rather than just pain and suffering more specifically will become obvious in Section 2. As we will show, slaughter cannot meet this criterion and so can never be truly considered humane, though different practices can still be better or worse on this dimension.

Assessing the humaneness of a practice involves “assessing what harms are done to the animals, how bad each harm is in terms of its intensity and duration, what methods are available or can be developed to minimise each harm, and the relative effectiveness of those methods of harm minimisation” ([2], s128). We must first look at how to remove or offset all harms, and then finally look at which harms remain and whether they are outweighed by the benefits of the practice. Although what we have left may not be a truly humane practice, it may still be a justifiable one if the benefits are sufficiently high, though the case for animal slaughter seems a weak one. We will discuss this further in Section 4.

The welfare issues surrounding the practice of slaughter are diverse and complex. They involve the capture and transport of animals from farm to abattoir, movement through the slaughterhouse, and of course, the actual process of stunning and killing itself. Attention to welfare concerns at all stages of this process is crucial if slaughter is going to be ‘humane’, in that it causes no (or minimal) harm to the animals. Although humane slaughter is commonly discussed within the animal welfare literature, the primary focus has been on the processes of slaughter and how to refine these to minimise welfare harm, rather than on the deeper question of the humaneness of slaughter itself. The underlying assumption appears to be that of course humane slaughter is possible, even if it may not always be achieved or carried out so in practice. Grandin [7], for instance, considers it possible for slaughter to be a form of euthanasia—i.e., a ‘good death’. In fact, confidence in the ability of abattoirs to perform humane slaughter has even led to calls for horse owners to send their horses destined for euthanasia to abattoirs instead, for cheaper and more humane killing [8].

There are a number of commonly used slaughter methods for agricultural animals [3,9]. These typically involve the use of a stunning method to render the animal unconscious, followed by the actual killing method. There are three primary types of stunning used—captive bolt, electrical and atmospheric. Captive bolt stunning is generally used for large animals, such as cattle. Here, an explosive force is used to drive a bolt into the skull and knock the animal unconscious (many captive bolt stunners are also designed to drive through the skull into the brain and cause brain death). When performed properly, this is an effective method of stunning (the latter more so than the former). Electrical stunning is often used for pigs, as their skulls make captive bolt stunning difficult. It is also used for chickens, who are hung upside-down by the legs and then passed down a line, in which their heads are passed through an electrified tank of salt water which paralyses and possibly stuns them. Atmospheric stunning involves the use of high atmospheric CO_2_ concentrations to induce insensibility through asphyxiation. Once stunned, animals are then hoisted by a leg and bled out through a cut to the throat. There are welfare concerns about all stages of these processes, particularly in the effectiveness of rapid stunning to ensure insensibility during killing, and thus, minimisation of pain and distress. These concerns have been well-covered elsewhere (e.g., [3,9,10,11]) and will not be the focus here.

One way we may deny the possibility of humane slaughter is by taking an alternative ethical position. There are two primary ethical positions regarding our treatment of animals—commonly referred to as the ‘welfare’ and the ‘rights’ positions [12]. The welfare position, based in utilitarian ethics, holds that our duties towards animals are to ensure their welfare. This allows for human use of animals in cases where they are provided with good welfare, or where the suffering inflicted on them is outweighed by other benefits, as per a typical consequentialist calculus. By contrast, the rights position—based in deontological ethics—holds our duties towards animals as being to uphold particular rights, such as rights to particular types of treatment, to liberty and to life. This view typically prevents most human uses of animals, as even where their good welfare can be assured, rights to life and liberty are still violated, and these cannot be offset through any benefits to others.

The very discussion of the possibility of humane slaughter presumes an animal welfare ethic, in which at least some forms of human use of and killing of animals may be permitted if they do not inflict unnecessary suffering. In this case, our focus would not be on the fact of death itself, but on the conditions during and prior to killing. By contrast, taking an animal rights stance would entail that there is no possibility of slaughter being humane in the context of using animals for human ends. The only form of killing that could be condoned would be that which is in the animal’s own best interests, in cases where we are ending ongoing suffering—what is commonly referred to as euthanasia (a similar discussion arises for the practice of management euthanasia in zoos [12,13]). This is certainly true, however to invoke the animal rights position in this way acts to stop the discussion before it even starts. There can no longer be engagement with those within the welfare position due to disagreement about the fundamental ethical framework. Though Pendergrast [14] may be correct that the rights position has been neglected within popular discourse surrounding animal slaughter, in this paper we will discuss slaughter from within the welfare position. Even from within this framework, it can still be the case that slaughter is not humane as it always negatively impacts welfare.

The paper will proceed as follows. In Section 2, we will argue that slaughter will always be at least partially inhumane, as though we can potentially remove the sources of pain and suffering accompanying the slaughter process, the animals will always have reduced welfare resulting from the deprivation of opportunity for future positive experiences. In Section 3, we will discuss the welfare impact of the ‘shape of a life’ (i.e., the distribution of various positive and negative experiences across the lifespan) and how harms inflicted at the end of life—such as those associated with slaughter—may be worse than those for which the animal has opportunity to outweigh with future positive experience. Finally, in Section 4, we will conclude with an examination of the implications of our discussion for research into and practice of animal slaughter.

## 2. The Harm of Death

Discussion of the welfare harms in slaughter typically cover the pain and suffering experienced by animals during the processes of transport, handling and killing. By contrast, here we argue that even if it were possible to design a slaughter process that caused none of these active harms to the animals, their welfare can still be harmed through the loss of life. Those within the welfare position have not usually considered death to be a welfare issue. This may be because welfare has typically been considered at a moment in time, rather than over the lifetime [15], or because welfare is typically taken to be experiential, such that those things which an animal cannot experience (such as death) cannot impact it [16]. However, both these assumptions can be challenged.

Welfare should not just be considered at a moment in time, but over a lifetime, and thus, consideration of future experience is important. Additionally, animal welfare does not include only the prevention of negative experiences such as suffering, but also the promotion of positive experiences, such as pleasure. Welfare should not just be considered good when suffering is reduced or removed, but also when pleasurable states are promoted. From this basis, we can differentiate, as per Regan [17], between welfare ‘harms’ (the direct creation of conditions that cause negative experiences for animals), and ‘deprivations’ (the removal of possibilities for positive experiences). Both of these conditions will decrease the welfare of an animal, as compared to what it otherwise might have been.

The combination of these two perspectives leads us to an understanding of why death is a welfare issue—because it removes the possibility for future positive experiences. If we aim (and we should) to maximise welfare, this means ensuring a minimum of negative experiences, and a maximum of positive experiences, over a lifetime. An animal that fails to meet these standards can be considered to have reduced welfare, compared to what it could otherwise have had were they met. This view, that a state of affairs is bad for an animal where it causes welfare to be lower than it otherwise might have been, or good if it causes it to be higher, is known as ‘comparativism’ [18].

The premature ending of life is bad for animals for precisely these reasons [12]. Welfare is not just considered for a moment in time but as the experiences of an animal over its lifetime, and includes positive as well as negative states. Thus, the shortening of life decreases welfare as it removes the possibilities for future positive experiences. This is cashed out by Wills [4] as the ‘deprivation account’ of the harm of death: “death is harmful for an individual if it makes that individual’s lifetime well-being lower than it otherwise would have been” (p. 29). In this way, slaughter will always fail to be truly humane in the strong sense we stated earlier, as it necessarily decreases lifetime welfare, and thus, is a comparative harm. Indeed, for these reasons, Wills [4] argues that killing should be legislated as a welfare harm, as it deprives animals of future potential positive experiences, such that individuals could be prosecuted for the act even when it does not inflict any suffering in the process.

Kasperbauer and Sandøe [19] argue additionally that premature killing harms animals by reducing their normal length of life. Many views of welfare take the ability to lead a normal, or natural, life, in accordance with species norms, to be a key component of welfare. This includes performance of species-typical behaviours, but can also include longevity. Under these views, killing will harm welfare in this way: “Insofar as natural living is indeed considered relevant to welfare, we predict that length of life—and thus killing animals—will become even more important to debates over animal welfare” ([20], p.31). Under this view, as well as removing future positive experiences, premature death could harm welfare through prevention of opportunity for species-typical longevity.

There are a number of potential objections to the view that slaughter is a welfare harm. The first is that the account relies on the deprivation of future positive experiences, but perhaps the animals taken to slaughter are those for which there will be no future positive experiences. Given the intense suffering that accompanies many intensive farming practices, animals coming from these systems may in fact be better off dead than alive. For example, broiler chickens are bred for rapid growth, which leads to them developing significant painful leg problems in trying to support this weight, and they are stocked in high densities in indoor barns, with poor air quality and unnatural light cycles, without any form of environmental enrichment [21]. Farmed sows are often housed in gestation crates with hard slatted floors, meant to protect the piglets, but which prevent the sow from turning around or lying down comfortably, and without any opportunities for mental stimulation [21]. 

These animals may have ‘lives not worth living’ (i.e., containing more negative than positive experiences) [22] and thus, slaughter could be seen as a benefit rather than a deprivation. This is in the same way that euthanasia is considered best for sick and injured animals with no prospect of recovery, where they are being spared future suffering. Although of course there is still a huge ethical issue to do with the suffering inflicted by these systems, this is not directly relevant to the question of slaughter. For these animals, slaughter could be a benefit, rather than a deprivation, and thus, would be humane.

There are two possible lines of response to this objection. The first is to deny that many animals actually experience lives that are so bad that they are not worth living, even within the worst intensive farming system. There are, after all, still possible pleasures available in the form of food consumption or interaction with social companions. However, this is not particularly convincing, when we look at the conditions of the worst of such systems. Instead, we can take a second line, and that is to emphasise that it is the reduction in possible future positive experiences that we are concerned with. Though these animals would not, in actuality, have good lives in the future, this is not a necessary feature of their lives. It is not a function of their intrinsic properties—in the way that euthanasia of a sick or dying animal is—but instead of their environment. There is the possibility for a good life for these animals, as evidenced by the positive experiences of ‘rescued’ ex-agricultural animals in sanctuaries, free to live out their days in comfort. Indeed, were we to take this objection seriously, then it seems that our moral duties to these animals are not to kill them as quickly as possible once they are brought into a life of suffering, but to act to prevent them being brought into this existence in the first place.

This links to a further potential objection that we would like to assess, which is the argument that these animals are only bred for agricultural purposes, and therefore, it is necessary that they are killed as a part of this life cycle. The claim here is that without the slaughter, they would not otherwise exist; therefore, they could not really be considered to be harmed by being slaughtered, as the alternative is nonexistence. At least they were able to live however many months or years they would not otherwise have had. This is a novel version of the non-identity problem, introduced by Schwartz [23] and famously discussed by Parfit [24]. Nevertheless, we consider this objection unconvincing (a similar criticism has been raised against genetic engineering and is similarly unconvincing [25] and we make an analogous argument against the objection here).

It relies on the concept of nonexistence as a harm, one that is worse than a shortened life. However, nonexistence is not a state and is not a harm. There is no creature that is being harmed by not coming into existence. By contrast, an animal that has been brought into existence is then one which can be harmed or benefitted, and this animal is harmed through premature loss of life. Although it may have been bred for the purpose of killing for food, this does not mean that it is not against its interests to be killed in this way. Imagine a former professional ballerina who artificially inseminates herself in order to create a child that will continue her dancing career. This career path causes a lot of pain and suffering, and we are indeed justified in criticising the mother, even though she would not have had the child if she had thought that her daughter would not continue her path. The same applies to farmed animals—the reason for being brought into existence cannot justify harm once created.

Further, even if it were the case that animals can be benefitted by being brought into existence at all, it seems that equally they could be harmed by this act [26]. In these cases, given the suffering caused throughout the life of these animals, it seems that their very existence is a harm and the industry they are a part of should not continue. Consider the related issue of de-extinction. If we were able to bring mammoths or other animals back into existence, the non-identity problem suggests that these animals cannot be considered harmed, if de-extinction involves necessary harms. Nevertheless, the suffering is real and morally relevant, and thus, should make us wary of the welfare these animals would experience [27]. It cannot be considered humane to create beings if such acts result in bad lives for the animals involved. They are in no way better off for being allowed to live whatever short lives they have, on the condition that they will eventually have to be killed (and, as above, the objection that killing in these cases will benefit them is weak).

A related objection comes in the form of the ‘replaceability argument’. That is, that the killing of one animal may free up space for another animal to come into existence that will then be the bearer of equal welfare, and thus, there is no net loss and no issue with the killing. As agricultural production is a constant cycle of breeding and slaughter, the replaceability argument holds particularly strongly in this industry. However, although this argument, if it holds (and see [28] for reasons to think that it does not), merely tells us that it is not all-things-considered worse to kill one animal if a replacement is brought into existence. It does not tell us that it is not worse for that animal, and as we have seen, it definitely is. Thus, this does not bear on the question of whether or not slaughter is humane, though it may then bear on the question of whether it is permissible, which we will return to in Section 4.

Another counterargument to our view comes from Belshaw [29], who argues that though death is bad for an animal, it is not bad in a way that matters morally. Much as lack of oil is bad for a tractor, we do not feel it creates any moral duties in us, so too is the death of an animal. He argues that it is only the desire for future life that makes death matter morally, particularly when it thwarts our plans and projects. As animals lack a conception of themselves as a being persisting through time, they cannot have such preferences for their future existence. At best, their desire for a continued future is instrumental—they may desire future experiences for the pleasure they create, but not continued life itself.

This is similar to a claim that animals can only be harmed by death if they possess the ability of ‘mental time travel’—a capacity to understand oneself as a being existing through time, to remember oneself in the past and imagine oneself as continuing into the future. The capacity for mental time travel may have selective advantages [30] but can also influence the welfare experience of animals. Animals that are able to remember their past will have a lasting welfare impact arising from past events; though, as Mendl and Paul [31] argue, the inability to remember does not mean past events do not impact welfare at all—they can still do so through lasting cumulative changes to learning and stress-response systems. Animals that are able to anticipate the future will benefit from anticipation of future events and have a stronger interest in their continued life. Without such an ability to project into the future, animals will be harmed less by their death than if they were able to do so, and thus, to desire their continued existence. 

This claim of course relies on empirical facts about animal psychology, and thus, is only as strong as our understanding of animal minds. Though Suddendorf and Corbalis [30] argue that current evidence for mental time travel in animals is inconclusive, they do not rule out the possibility of at least some animals possessing this ability; Mendl and Paul [31] argue that the abilities animals do possess—‘recollection-like experiences’—are probably sufficient to create some welfare impact, at least in regards to past events. However, there is currently little to no evidence that animals possess the ability to mentally time travel into the future or to hold preferences about their own continued life. If they did, then we could say that their deaths matter in the way required by Belshaw.

Similarly, one further way in which slaughter can be a harm is if animals possess a concept of death and are able to fear it or specifically desire continued life. It is currently unclear whether animals possess such an understanding but Monsó [32] provides an outline of how we might go about finding out. For now, we will remain agnostic on the question, but simply flag here that if it were the case—at least, if animals were able to understand the fact of their own upcoming death—it would actually further increase the harm of slaughter. Until we know more about animals, it might be premature to accept that they lack this understanding.

However, there is a stronger conceptual response to this counterargument by Belshaw, and that is that the loss of opportunities for positive welfare actually are the sorts of things that should matter morally. Taking away an opportunity for me to experience some form of pleasure will still decrease my welfare, regardless of whether or not I was aware of the opportunity and desired it, as I now have lower welfare than I might otherwise have had. This is relevantly different than the case of tractors, or plants, as these objects lack the experiential component of good or bad events that make the events matter to them [33]. It is the fact that these experiences matter to the animals themselves that adds moral weight to their experiences, regardless of whether or not they are able to desire them in advance.

As we have argued, slaughter necessarily shortens life, and thus harms overall welfare due to deprivation of opportunity for future positive experiences. As slaughter is always a harm to welfare, it can thus never truly be humane. We will turn in Section 4 to some implications of this, but first we will discuss another way in which slaughter can be seen as a welfare harm.

## 3. Shape of a Life

There is another way in which slaughter can be an additional welfare harm, and this is a result of its positioning at the end of the life. One concern when considering the welfare of animals is what is known as the ‘shape of a life’—the distribution of various positive and negative experiences across the lifespan: “the temporal sequence of good and bad times in a life can be a valuable feature of that life as a whole” ([34], p.305). We established in the previous section that it is not animal welfare at a specific time (synchronic welfare) that matters, as much as it is welfare over a lifetime (diachronic welfare). The shape of life can be thought of in these terms as well, as the distribution of synchronic welfare, or the ‘shape’ of this curve [35]. Intuitively, most people feel like (all other things being equal) a life that begins poorly and ends well is better than a life that begins well and ends poorly; there is value in an upward trend and disvalue in a downward one [36].

This seems to contradict two parts of typical thinking about welfare, the first that lifetime welfare is an additive sum of momentary welfare experiences (intralife aggregation) and the second that something is valuable regardless of its temporal location (temporal neutrality) [34]. On the first, most views of animal welfare are aggregative, in which lifetime welfare consists of the sum of positive and negative welfare experiences across that lifetime [37]. However, within an aggregative view like this, it is obvious that the temporal location of these experiences within the life will make no difference to welfare overall. Indeed, temporal neutrality is often taken to be a core tenet of rationality—we should not prefer to have our pleasures now, rather than in the future, for instance [34] (unless there is a risk of early death, in which case one could discount future pleasures accordingly). However, if we take seriously these intuitions about the shape of a life, we cannot take welfare to be solely the sum of momentary experiences with no reference to their temporal location.

One way we might explain the shape of life intuitions is through their interaction effects—having one experience prior to another may create anticipation effects or expectations that will affect how the second is experienced, and thus change the welfare impact. This would make the shape of life instrumentally, rather than intrinsically, valuable [34]. For example, enclosure space is an important determinant of welfare for captive animals [38]. It is likely to be worse for an animal to have the positive experience of a larger enclosure space before the negative experience of a smaller one, because the perceived decrease in quality will add to the negative feelings brought about by the change. Conversely, moving from a smaller to a larger space will create additional positive feelings about the improvement. Therefore, it is not just the number of positive and negative experiences that influence total welfare, but the order of and interactions between them. However, these are not quite the type of cases we are interested in. We are interested in cases where, even if the degree of impact remains constant, the order may still be important. This view is that it is intrinsically bad to be worse off than you previously were, in addition to simply the effect of the bad event itself; the reverse is true for being better off than you were previously—it is the trend itself which holds value [39]. Losing is itself a disvalue and gaining is a value.

The problem that the shape of a life hypothesis creates for the case of slaughter is that slaughter is a negative experience that necessarily occurs at the end of life. Slaughter is always going to be a negative experience for animals. Despite even the best efforts to ensure humaneness of the slaughter process, there is inevitably some associated stress and suffering. Indeed, current abattoirs typically function at far less than best standards [9], and a large number of animals can undergo a serious amount of pain and suffering during the final moments of their lives. For example, take fish, who are receiving increasing attention as regarding their welfare, as current scientific evidence is in favour of the sentience of fish [40,41] (in a similar vein, octopus farming has recently gained attention in both popular media and academic circles; with octopuses also being recognised as sentient, there are calls for legislators to reconsider whether current octopus farming and slaughter practices can be considered humane [42,43,44]). The number of farmed fish importantly also far outweighs that of other vertebrates [45]. Fish are often slaughtered using asphyxiation in air or on ice, which can take several hours and during which the fish appear to retain consciousness [45]. Similarly, for immersion in water high in CO_2_, fish show signs of distress as they die [45]. They can also be bled or beheaded while conscious, or first stunned using percussive or electrical methods, with varying degrees of success depending on correct application [45]. In cases of other animals, oftentimes the stunning process fails, and the slaughter and processing procedures can occur while animals are still conscious [1]. This can be a result of mistakes due to the speed of processing or lack of worker training. Additionally, there are frequent reports of deliberate cruelty towards the animals within slaughterhouses [1]. Studies of abattoir workers have shown that the mental and emotional strains of the job often lead to coping mechanisms such as compartmentalising or decreasing their emotional connection to animals, which can lead to increased neglect and cruelty [46].

Animals going to slaughter are thus experiencing a (usually strong) negative right at the end of their lives. This seems not just instrumentally bad, as even when animals have no anticipation or expectation of their upcoming death or suffering such that the order matters, or are unable to lament their fate based on the decline from previous high points, it still intuitively feels like ending life with suffering is worse than experiencing it at some earlier time. This intuition may be in part because, unlike other negative experiences, slaughter does not hold the possibility of a future positive experience that can offset it. In other cases, an episode of suffering can be somewhat compensated by a future positive. If we undergo a painful medical procedure but then are able to go out the next day for our favourite meal, there is a sense in which the positive event goes some way towards overwriting the prior negative event. In cases of slaughter, this is never possible, and thus, the negative experiences seem to be even worse. In this sense, because slaughter necessarily creates negative experiences at the end of life, it creates additional welfare harm, and thus, is less humane for this reason also.

## 4. Implications for Animal Slaughter Practices

In this paper, we have shown that there are problems with even the best practiced slaughter. Due to the deprivation inherent in loss of life, slaughtered animals will always have reduced welfare. Additionally, the infliction of suffering at the very end of life is problematic, as it does not allow for offsetting with future positive experiences. For these reasons, no slaughter will be truly ‘humane’ in the deeper sense we established in Section 1, i.e., slaughter without harm to welfare. In this section, we will discuss the implications of this perspective for the practice of animal slaughter.

Mellor and Littin [2] list some commonly accepted ethical principles applying to the use of animals. These include: “People should not harm animals unless it is absolutely necessary; If there are less painful or distressing ways of treating animals they should be used; [and] Some harms should be prohibited, regardless of their benefits” (s127). These are relevant to the question of humane slaughter. In particular, the first and third imply that perhaps, if slaughter is harmful, then it should not be permissible, as it should not be considered as ‘absolutely necessary’. The second tells us that even if not prohibited, we should still make an effort to minimise the harms. Although it may not be possible at this stage to prevent some of the harms of slaughter—those discussed within this paper—we can still minimise others, such as pain and fear.

In the first instance, we might say that the fact that slaughter can never be humane means that it is impermissible. This could then lead to campaigning for cessation of the practice and, by extension, the agricultural industry that relies on it. As argued by Pendergrast [14], dissenting voices in the debate are often silenced, so that the question becomes simply how to slaughter more humanely, rather than whether we should slaughter at all. In this paper, we have come from within the animal welfare position to provide support for the view that naturally arises from the rights position: that animal slaughter is essentially problematic, and hence, can never be humane. With the increasing popularity of plant-based food items, perhaps this larger change is not entirely unrealistic in the longer-term future. However, this will not help animals now, who can still experience improvements.

Conversely, there is also the possibility to argue that even if slaughter is not humane in the stronger sense that we are advocating, this does not make it impermissible. Instead, we might think that it is only the strongly inhumane practices, those that inflict suffering, that are impermissible. Those which harm welfare through deprivation may be dis-preferred, but still acceptable, particularly in cases of greater benefit. This is also supported by a view of the asymmetry of pleasure and suffering—that prevention of suffering carries more moral weight than the provision of pleasure [47]. Though the provision of pleasure matters morally, it does so to a much lesser degree than the prevention of suffering. Thus, the harm of slaughter, in terms of deprivation of future pleasures, is a much lesser harm than the suffering inflicted during the process and attention to improving these techniques could be seen as more valuable than campaigning for their end.

Either way, our arguments for the inability for truly humane slaughter should not be seen to undermine the importance of the research being done in this area or the push to improve practices. While slaughter may always be at least somewhat inhumane, it can be more or less so. If we cannot prevent it, we should at least aim for improvement. Particularly when considering the shape of life, the best way to decrease this problem is to continue research and development of slaughter practices and techniques, such that it contains a bare minimum of suffering.

There are several ways in which improvements in slaughter practices can be driven, including scientific, economic and legislative. As mentioned in the beginning, there is a lot of work in animal welfare science aimed at investigating and improving slaughter. Scientific inquiry into the specific degree of harm inflicted by particular slaughter and pre-slaughter practices, and development of alternative practices which are more humane, will serve to inform and motivate consumers, producers and legislators to enact change.

The biggest problem with the slaughter process is that it is necessarily constrained by economic concerns of cost and convenience [45]. Concerns for animal welfare play a secondary role and changes will only be implemented where they are cost-effective. However, the increasing concern from consumers towards the welfare of animals at all stages of the production process [10] can help drive demand for such changes and make them economically viable. It has historically been rising consumer awareness of the practices of slaughter that has driven calls for improvements in welfare, such as the introduction of the Humane Methods of Slaughter Act in the USA in 1958 [48]. Consumer demand can help lead changes in industry practices, as well as in legislation covering slaughter.

Legislation plays an important role in the regulation of animal slaughter [9]. The practices used in slaughterhouses typically conform to the minimum standards required by law, as to implement higher standards would be to incur unnecessary costs. Though, in many cases, violations of even these minimum standards can be found in slaughterhouses for a number of reasons [9]. Primarily these are due to what is often a low degree of monitoring and enforcement. Human error or deliberate mistreatment are also relatively common. Exemptions for particular animal groups, such as poultry or those used in religious ritual slaughter, are also the source of welfare problems. Campaigning for improved enforcement of existing laws would assist in ensuring the highest standards of animal welfare during the slaughter process.

## 5. Conclusions

While the practice of slaughter continues, all effort should be made to ensure that animal welfare remains as high as possible. This obviously involves the removal or minimisation of pain and/or suffering, but perhaps even some consideration of positive welfare states could be introduced (here, the rise of animal sentience research—such as the recent establishment of the journal *Animal Sentience*—has played an important role in understanding the mental and emotional lives of animals and their link to welfare [49,50]). It may sound a somewhat absurd suggestion, given the current state of the practice and the amount of suffering yet to be eliminated. However, there is no reason that slaughterhouses could not, in theory, provide some positive experiences for animals during the pre-slaughter period, such as pleasant sounds, smells and activities. For instance, the more intelligent an animal, the more cognitive stimulation it requires in order to avoid boredom and experience positive states such as pleasure and excitement [51]. This may then, in the end, create something more akin to a companion animal quietly drifting away in the arms of a loving owner than the current industrialised abattoir procedures. This would thus make the end of life less negative and offset some of the shape of life considerations. Although we have argued that slaughter will always cause some harm, and thus, never be truly humane in a deep sense, we still support any and all moves to improve welfare and move closer to a more humane slaughter process.
